# Proteome-Wide Analyses Reveal the Diverse Functions of Lysine 2-Hydroxyisobutyrylation in *Oryza sativa*

**DOI:** 10.1186/s12284-020-00389-1

**Published:** 2020-06-05

**Authors:** Chao Xue, Zhongying Qiao, Xu Chen, Penghui Cao, Kai Liu, Shuai Liu, Lu Ye, Zhiyun Gong

**Affiliations:** 1 Jiangsu Key Laboratory of Crop Genetics and Physiology/ Key Laboratory of Plant Functional Genomics of the Ministry of Education/ Jiangsu Key Laboratory of Crop Genomics and Molecular Breeding, Agricultural College of Yangzhou University, Yangzhou, 225009 China; 2 Jiangsu Co-Innovation Center for Modern Production Technology of Grain Crops, Yangzhou University, Yangzhou, 225009 China; 3 Suzhou Academy of Agricultural Sciences, North of Wangting Town, Suzhou, 215128 China

**Keywords:** *Oryza sativa*, Lysine 2-hydroxyisobutyrylation, Photosynthesis, Histone acylation

## Abstract

**Background:**

Lysine 2-hydroxyisobutyrylation (Khib), a newly identified post-translational modification, is known to regulate transcriptional activity in animals. However, extensive studies of the lysine 2-hydroxyisobutyrylome in plants and animals have yet to be performed.

**Results:**

In this study, using LC-MS/MS qualitative proteomics strategies, we identified 4163 Khib sites on 1596 modified proteins in rice (*Oryza sativa*) seedlings. Motif analysis revealed 10 conserved motifs flanking the Khib sites, and subcellular localization analysis revealed that 44% of the Khib proteins are localized in the chloroplast. Gene ontology function, KEGG pathway, and protein domain enrichment analyses revealed that Khib occurs on proteins involved in diverse biological processes and is especially enriched in carbon metabolism and photosynthesis. Among the modified proteins, 20 Khib sites were identified in histone H2A and H2B, while only one site was identified in histone H4. Protein-protein interaction (PPI) network analysis further demonstrated that Khib participates in diverse biological processes including ribosomal activity, biosynthesis of secondary metabolites, and metabolic pathways. In addition, a comparison of lysine 2-hydroxyisobutyrylation, acetylation, and crotonylation in the rice proteome showed that 45 proteins with only 26 common lysine sites are commonly modified by three PTMs. The crosstalk of modified sites and PPI among these PTMs may form a complex network with both similar and different regulatory mechanisms.

**Conclusions:**

In summary, our study comprehensively profiles the lysine 2-hydroxyisobutyrylome in rice and provides a better understanding of its biological functions in plants.

## Background

Posttranslational modifications (PTMs) of proteins play important roles in metabolic regulation, protein interaction, cell signaling, and other biological processes (Choudhary et al. 2014; Martin and Zhang 2005). In recent years, increasingly novel types of PTM on lysine residues have been identified and demonstrated to be associated with regulatory functions. These PTMs include lysine succinylation (Ksucc), butyrylation (Kbu), propionylation (Kpr), crotonylation (Kcr), malonylation (Kma), β-hydroxybutyrylation (Kbhb), and glutarylation (Kglu) (Chen et al. 2007; Tan et al. 2014; Xie et al. 2012; Xie et al. 2016; Zhang et al. 2011; Tan et al. 2011). Like acetylation (Kac), these emerging PTMs are classified as lysine acylations and have been demonstrated to occur on multiple proteins involved in various cellular metabolic processes (Lin et al. 2012; Weinert et al. 2013). A growing body of evidence indicates that lysine acylation or deacylation can be catalyzed by certain lysine acetyltransferases (KATs) and deacetylases (KDACs) (Huang et al. 2018a). Previous studies have revealed that p300, a lysine acetyltransferase, can catalyze other acylations besides lysine acetylation, including crotonylation, butyrylation, propionylation, and 2-hydroxyisobutyrylation (Huang et al. 2018b; Sabari et al. 2018; Chen et al. 2007). Furthermore, many histone acylations have been shown to exhibit functions in modulating chromatin status and stimulating transcriptional activity (Sabari et al. 2018; Tan et al. 2011).

Lysine 2-hydroxyisobutyrylation (Khib) is a novel PTM first identified in human and mouse proteins as a widely distributed active histone mark (Dai et al. 2014). In this pioneering study, histone Khib was shown to have a different genomic distribution from those of histone Kac or Kcr during male germ cell differentiation. The structural differences of Khib from lysine methylation, acetylation, and crotonylation enable it to induce a larger change in modified-lysine size, and the hydroxyl group of Khib allows the modified lysine to form hydrogen bonds with other molecules, which is important for regulating protein functions (Dai et al. 2014; Maxwell et al. 1999). H4K8hib is associated with active gene transcription in mouse meiotic and post-meiotic cells. Furthermore, H4K8hib has been demonstrated to regulate gene transcriptional activity in *Saccharomyces cerevisiae* and is differentially regulated under different glucose concentrations (Huang et al. 2017). Besides, Khib in non-histones has been also involved in diverse biological processes, including the tricarboxylic acid cycle, glycolysis/gluconeogenesis, and especially enriched in mitochondrial proteins within energy metabolic networks (Huang et al. 2018a; Wu et al. 2018; Huang et al. 2018b). These findings indicate that Khib has regulatory functions on cellular processes and gene transcriptional activity. However, the regulatory mechanisms of this new PTM in diverse biological processes are not well understood for both eukaryotes and prokaryotes.

In recent decades, the application of high-specificity pan-antibodies and high-sensitivity mass spectrometry (MS) techniques have broadened the current catalogue of PTMs on histones and non-histones. MS analysis relies on the identification of modification-induced changes in the masses of peptides, which are termed mass shifts (Sabari et al. 2017). Once a mass shift caused by a PTM is detected or localized to certain residues, the chemical structure of the modifier can be deduced and validated through high performance liquid chromatography (HPLC) co-elution, tandem mass spectrometry analysis, isotopic labeling, and immunochemistry using an appropriate antibody (Chick et al. 2015; Dai et al. 2014; Tsur et al. 2005). Accordingly, MS-based approaches have become highly efficient and powerful methods for large-scale analysis of PTMs and identifying novel lysine modifications.

For example, the first global profiling of the Khib proteome in mammalian cells identified 6548 Khib sites on 1725 substrate proteins (Huang et al. 2018a). By combining affinity enrichment with HPLC-MS, a total of 4735 lysine sites on 1051 proteins in *Proteus mirabilis* were found to be 2-hydroxyisobutyrated, indicating that Khib may play important roles in bacterial metabolism (Dong et al. 2018). Furthermore, proteome-wide analysis of Khib in *Physcomitrella patens* identified 11,976 Khib sites in 3001 proteins involved in a wide range of molecular functions and cellular processes (Yu et al. 2017). Together, these findings show that Khib may have diverse and novel functions on biological processes in eukaryotes and prokaryotes.

Rice (*Oryza sativa*) is an important model organism for cereal research and provides food for more than half of the world’s population (Ashikari et al. 2005). In recent years, rapid development of proteomics techniques and specific pan-antibodies have dramatically expanded the global profiling of the PTM proteome in rice, including the acetylome, succinylome, ubiquitome, crotonylome and 2-hydroxyisobutyrylome (He et al. 2016; Liu et al. 2018; Meng et al. 2017; Xie et al. 2015; Xiong et al. 2016; Xue et al. 2018).

Our previous study identified 1353 Kac sites on 866 proteins in rice seedlings and demonstrated that H3 (lysine 27 and 36) acetylation increased in response to cold stress (Xue et al. 2018). Furthermore, the first proteomic analysis of lysine crotonylation in rice identified 1265 Kcr sites on 690 proteins and proved that histone Kcr is positively correlated with gene expression in plants (Liu et al. 2018). In another study, a total of 699 acetylated sites on 389 proteins and 665 succinylated sites on 261 proteins were identified in the embryo of germinating rice seed using HPLC-MS/MS (He et al. 2016). More recently, a huge lysine 2-hydroxyisobutyrylome dataset with 9916 Khib sites on 2512 proteins was identified in developing rice seeds (Meng et al. 2017), providing the first systematic analysis of the Khib proteome and its potential regulatory functions for grain filling and development in rice. Like other acylations, Khib may have similar potential physiological functions on plant growth and development. However, the number of reports on Khib in plants is still very limited.

In this study, utilizing 2-hydroxyisobutyrylation affinity enrichment and LC-MS/MS qualitative proteomics strategies, we identified 4163 2-hydroxyisobutyryl lysine sites on 1596 proteins in rice seedlings. A detailed proteomic analysis revealed that Khib occurs in proteins involved in biological functions and metabolic processes. Through the crosstalk of the 2-hydroxyisobutyrylome, crotonylome, and acetylome in rice leaves, we found that these acylations have overlapping modified proteins and sites to participate in regulatory metabolisms, but also have significant distinctions to execute diverse protein functions.

## Results

### Proteome-Wide Analyses Revealed a Large Lysine 2-Hydroxyisobutyrylome in Rice

**Fig. 1:**
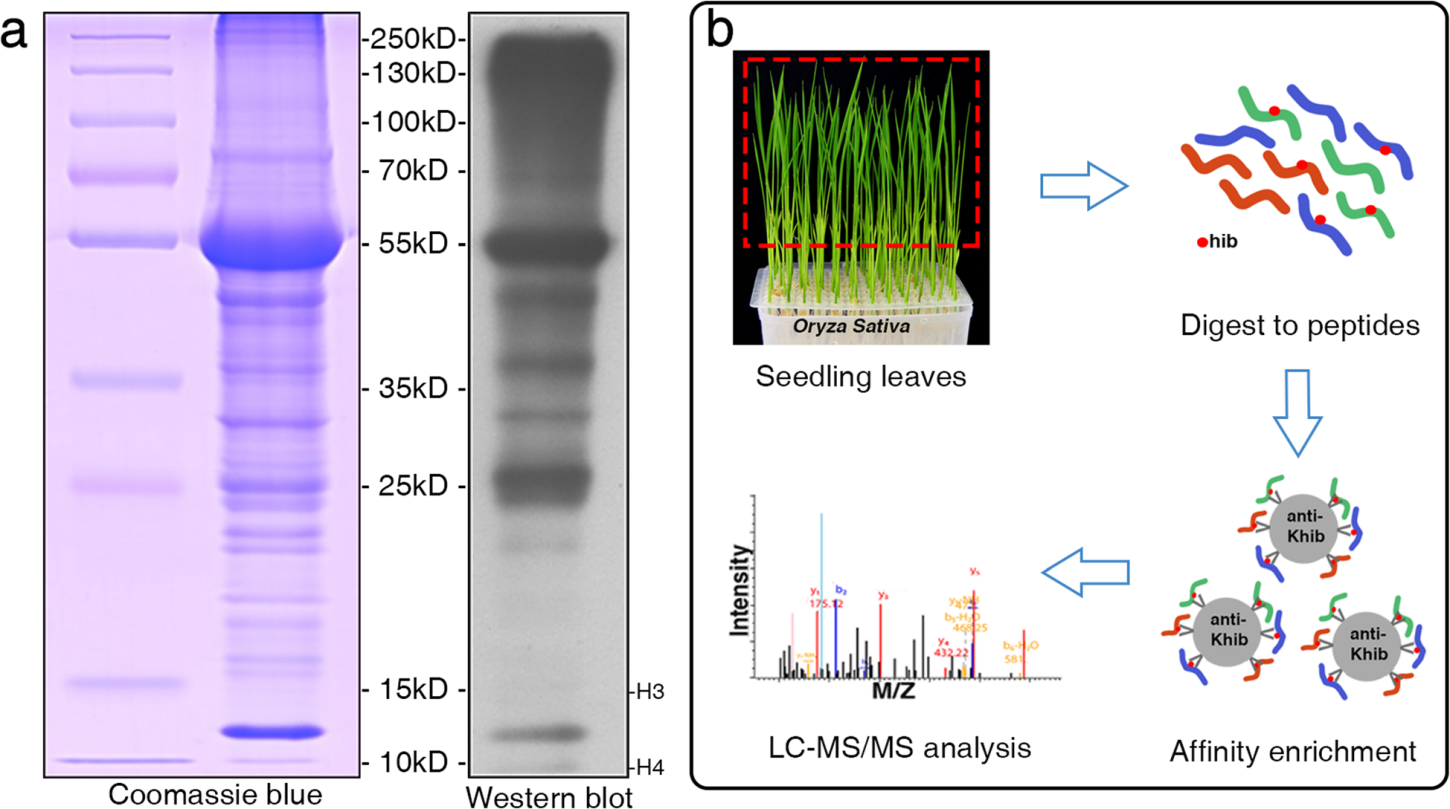
Workflow for large-scale identification of Khib sites in rice. **a** Western blotting analysis of total proteins in rice seedling leaves. The gels for SDS-PAGE were stained with Coomassie Blue. **b** Workflow for identification of Khib modification in rice. Total proteins extracted from seedling leaves were digested to peptides by trypsin. Peptides bearing Khib sites were affinity enriched with a specific Khib antibody and subsequently analyzed by LC-MS/MS.

Khib has been proved to be involved in diverse biological processes in eukaryotes and prokaryotes (Yu et al. 2017; Tan et al. 2011; Dai et al. 2014; Dong et al. 2018). To investigate the distribution of Khib modification in the proteins of rice, we performed Western blot assaying using pan − 2-hydroxyisobutyryllysine antibody (Fig. 1a). The sodium dodecyl sulfate polyacrylamide gel electrophoresis (SDS-PAGE) and immunoblotting results showed multiple bands from the leaf proteins, suggesting that Khib is widely distributed in rice seedlings. Furthermore, the blotting results displayed distinguished bands at 10–15 kD where the histones are located. However, no smear was observed at the position of histone H3 (~ 15 kD).

Furthermore, we combined affinity enrichment and high-resolution LC-MS/MS methods to elucidate the 2-hydroxyisobutyrylome in rice seedlings. Briefly, proteins isolated from seedling leaves were digested to peptides. Then, the peptides with Khib sites were enriched by Khib specific antibodies. Subsequent LC-MS/MS analysis was performed after affinity enrichment (Fig. 1b). MS data were validated by checking the mass errors of all the identified peptides. Most of the mass errors fell in the range of ±2 ppm, with most peaks less than 0.02 Da, demonstrating high mass accuracy (Additional file 1: Fig. S1a). Additionally, the length of most of the peptides was between 7 and 20 amino acids, which is in broad agreement with the expected tryptic peptide profile, further confirming the high quality of the sample preparation (Additional file 1: Fig. S1b). Of all the 4535 peptides detected, 4129 peptides (91.0%) with Khib were identified. In total, 4163 2-hydroxyisobutyryl lysine sites were identified on 1596 proteins in rice leaves (Additional file 2: Table S1). Furthermore, we found that 825 proteins (51.7%) were 2-hydroxyisobutyrylated at only one site, while a very low percentage (ca. 5%) were modified at more than eight sites (Additional file 1: Fig. S1c). Some example mass spectra of Khib-modified peptides on representative proteins are shown in Fig. S2.

### Identification of Khib Sites Reveals Conserved Motifs in Rice

**Fig. 2:**
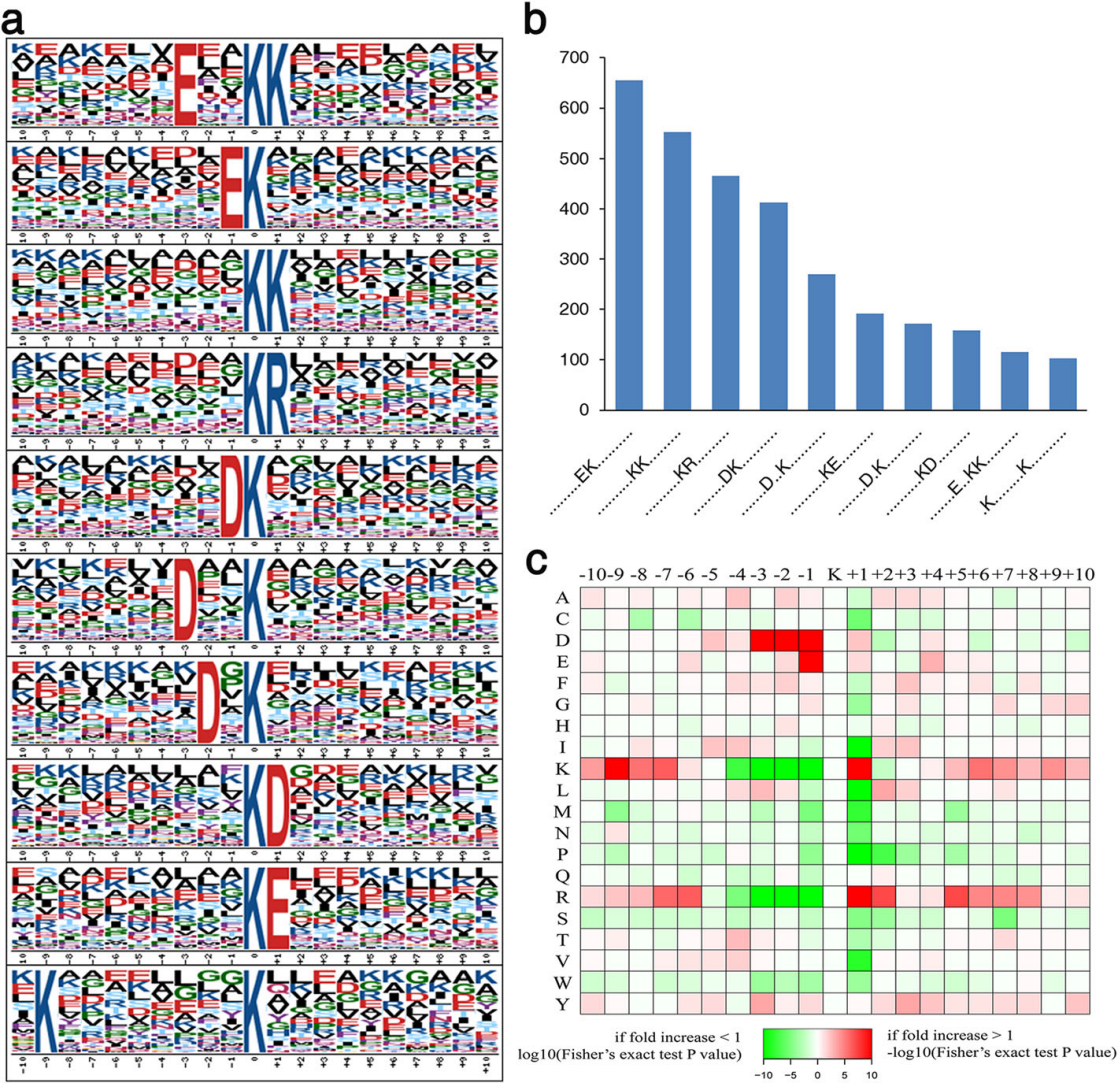
Motif analysis of all identified Khib sites. **a** Conserved motifs identified around the Khib sites. The size of each letter reflects the frequency of that amino acid residue in that position. **b** Frequency for each motif occurring in peptides with Khib modification. **c** Heat-map of the amino acid compositions of the Khib sites, showing the enrichment (red) and depletion (green) of amino acids in each position (from − 10 to + 10) flanking the Khib sites

To investigate the amino acid sequence motifs around the Khib sites, Motif-x software was used to discover conserved motifs from amino acid residues located within 10 amino acids upstream or downstream of the 2-hydroxyisobutyrylated lysine. Ten conserved motifs were detected in the rice 2-hydroxyisobutyrylome around Khib sites (Fig. 2a, b), such as EK_hib_, K_hib_K, K_hib_R, DK_hib_ and D_XX_K_hib_ (K_hib_ indicates the 2-hydroxyisobutyrylated lysine, and _X_ indicates a random amino acid residue). The motif patterns exhibit a preference for polar negatively charged amino acids, i.e., aspartate (D) and glutamate (E), flanking the modified lysine site. Similar amino acid sequence features have also been observed in the 2-hydroxyisobutyrylome of developing rice seeds (Meng et al. 2017). In addition, heat-map analysis of enriched and depleted amino acids showed that aspartate (D) at the − 1, − 2, and − 3 positions and glutamate (E) at the − 1 position are favorable for 2-hydroxyisobutyrylation (Fig. 2c). Furthermore, polar positively charged amino acids, i.e., lysine (K) and arginine (R), are overrepresented at the + 1 position. However, lysine (K) and arginine (R) at the − 1 to − 4 positions are underrepresented. Thus, these results indicate that Khib is preferred at lysine residues adjacent to polar hydrophilic amino acids.

### Functional Annotation and Subcellular Localization Analyses of Khib Proteins

**Fig. 3:**
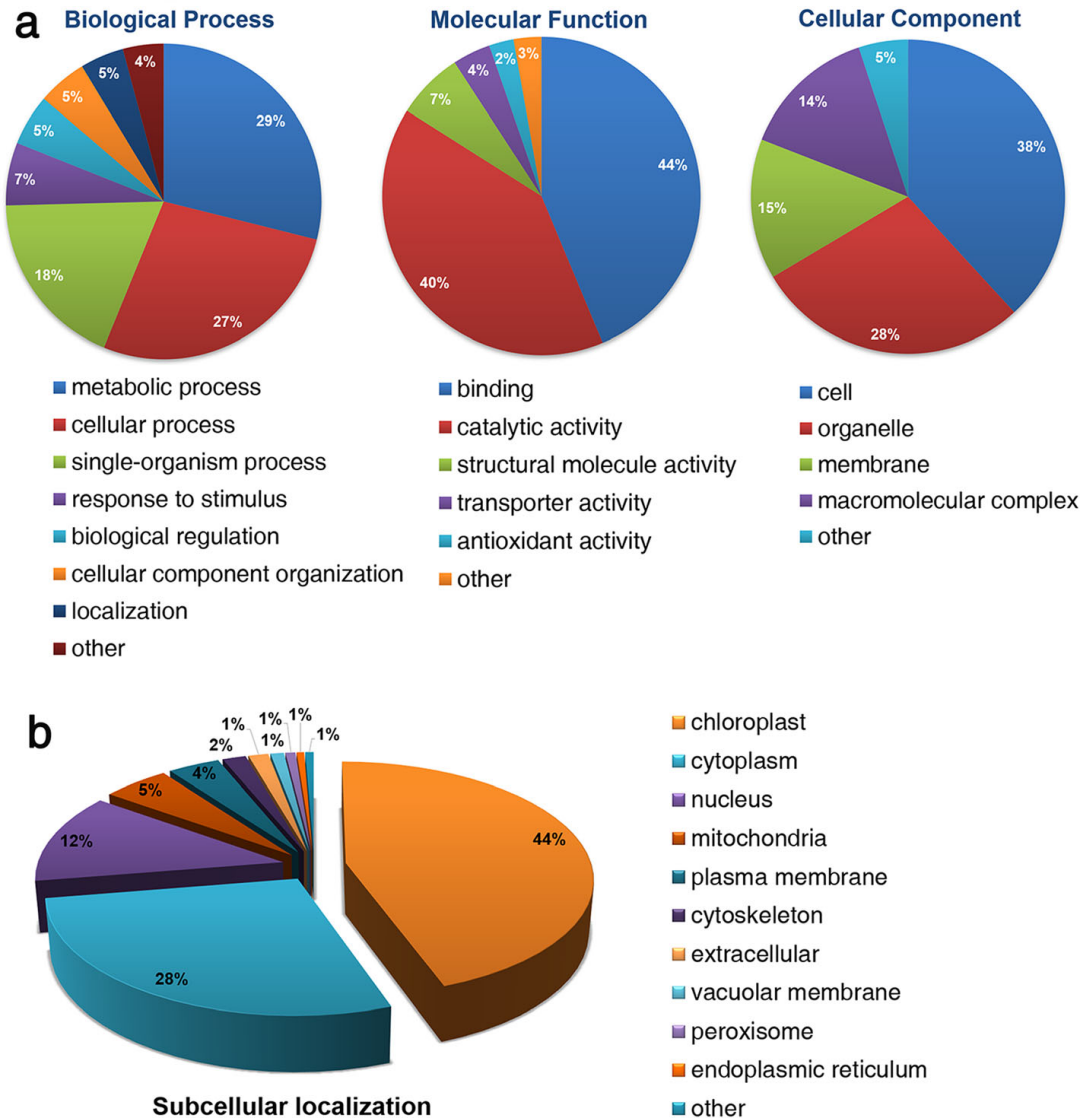
GO functional classification and subcellular localization of Khib modified proteins. **a** Classification of Khib proteins based on biological process, molecular function, and cellular component. **b** Subcellular localization of Khib proteins

Gene ontology (GO) analysis is typically used to ascertain gene or protein roles in eukaryotes and prokaryotes based on three independent ontologies, i.e., biological process, molecular function, and cellular component (Ashburner et al. 2000). Accordingly, to elucidate the function and distribution of Khib proteins in rice seedlings, GO classification and subcellular localization analysis were performed (Fig. 3). GO functional analysis revealed proteins mainly involved in metabolic (29%), cellular (27%), and single-organism (18%) processes and demonstrated that most Khib proteins are associated with binding (44%) and catalytic (40%) activity based on molecular function (Fig. 3a). Regarding the cellular component category, 38% of the Khib proteins were located in the cell, 28% in organelles, 15% in the membrane, and 14% in macromolecular complexes. The classification of Khib proteins is very similar to those that undergo other acylations in rice, such as acetylation (Ashburner et al. 2000; Meng et al. 2018; Xue et al. 2018), succinylation (He et al. 2016), and crotonylation (Liu et al. 2018), suggesting that various acylations may be involved in these molecular functions or biological processes.

The subcellular localization prediction for the modified proteins was performed using WoLF PSORT software (Fig. 3b). Most of the Khib occur in proteins located in the chloroplast (44%), cytoplasm (28%), and nucleus (12%). To verify the subcellular localization analysis, we performed Western blot in the chloroplast proteins with pan- 2-hydroxyisobutyryllysine antibody (Additional file 1: Fig. S3). The immunoblotting results indicate that a large proportion of 2-hydroxyisobutyrylated sites were located in the chloroplast proteins. Furthermore, the functional annotation and subcellular localization results indicated that Khib may have important biological functions in diverse metabolic processes and different cellular components.

### Function, Pathway, and Domain Enrichment Analyses of Khib Proteins in Rice

To investigate the metabolic processes in which the 1596 Khib proteins are involved, GO-based enrichment analysis was performed (Additional file 1: Fig. S4). The GO enrichment analysis revealed that the terms ‘unfolded protein binding’, ‘translation factor activity’, ‘oxidoreductase activity’, and ‘chlorophyll binding’ are most significantly enriched in the molecular function category. In terms of cellular components, Khib proteins are mainly enriched in the cytoplasm, plastid, chloroplast, and intracellular parts.

**Fig. 4:**
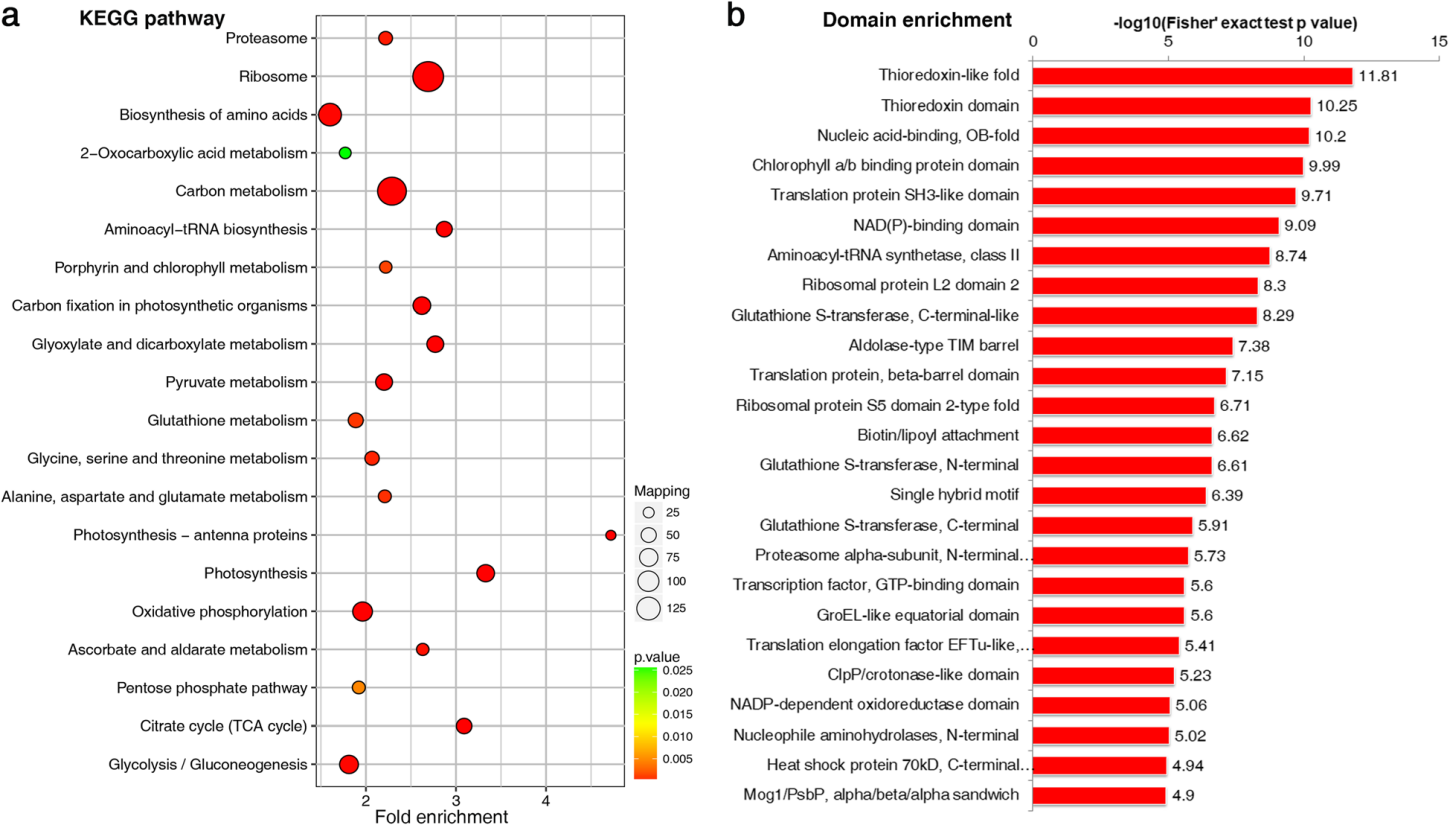
KEGG pathway and Protein domain enrichment analyses of 2-hydroxyisobutyrylated proteins

In terms of biological processes, the metabolic processes involving organonitrogen compounds, small molecules, carboxylic acids, organic acids, and oxoacids, as well as photosynthetic reactions, were most enriched. KEGG pathway enrichment analysis showed that proteins with Khib are involved in numerous central metabolic processes including photosynthesis, carbon metabolism, ribosomal activity, carbon fixation in photosynthetic organisms, and the citrate cycle (Fig. 4a; Additional file 1: Fig. S5). Furthermore, protein domain enrichment analysis identified 25 significantly enriched domains (Fig. 4b), including the thioredoxin-like fold, chlorophyll a/b binding protein domain, and NAD (P)-binding domain. Proteins with these domains are important in catalytic activity, photosynthesis, carbon fixation, and other central metabolic functions. Thus, enrichment analysis of Khib-modified proteins in rice further indicated that Khib plays pivotal roles in photosynthesis and carbon metabolism.

### Proteins Mostly Involved in Photosynthesis and Carbon Metabolism Are Modified by Khib

**Fig. 5:**
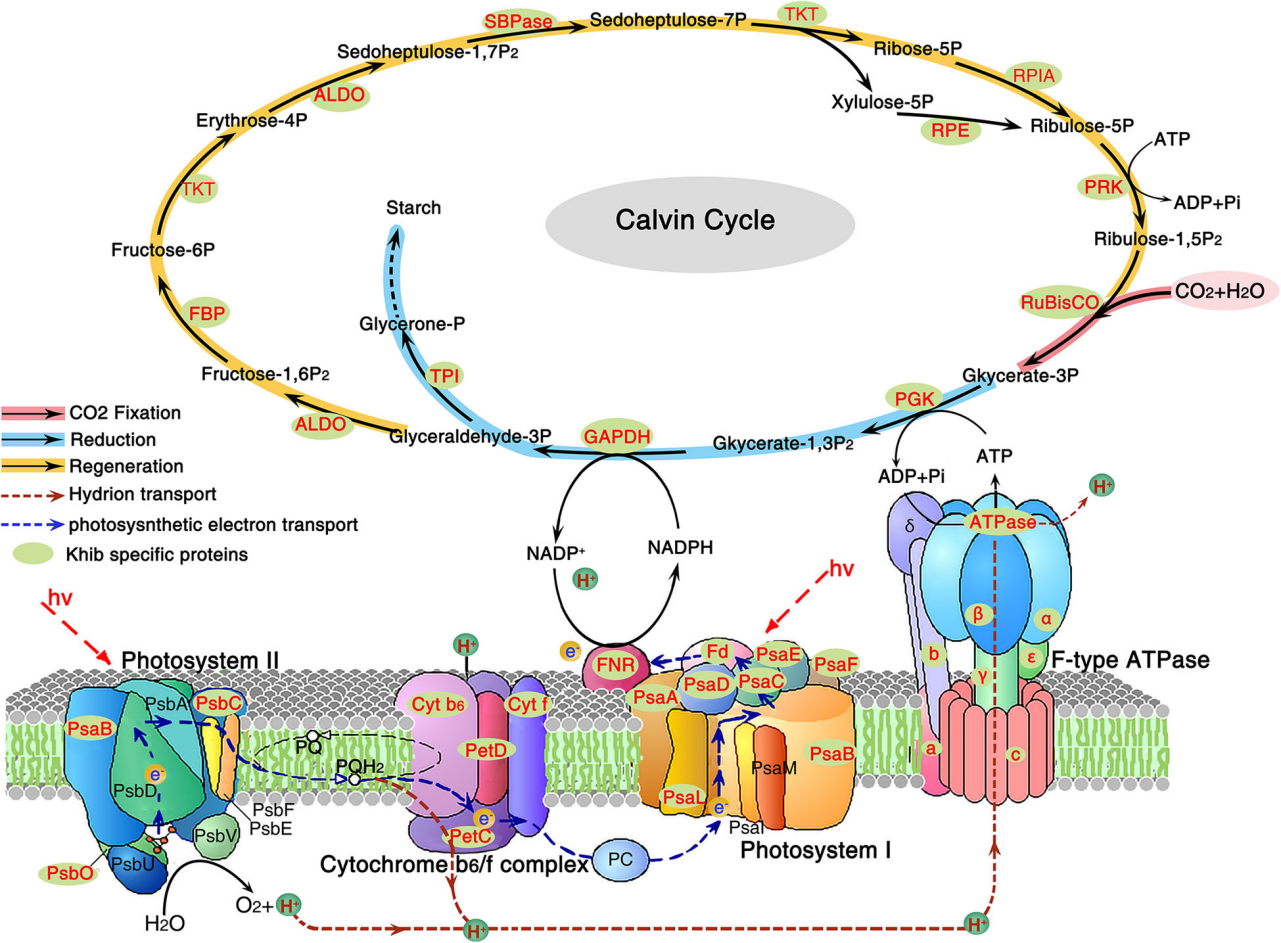
Khib proteins enriched in carbon metabolism and photosynthesis pathways in rice. The 2-hydroxyisobutyrylated enzymes or proteins identified are highlighted in red

According to the above enrichment analysis, we found that a large proportion of Khib-modified proteins are significantly associated with translation factor activity and chlorophyll binding functions, and they are enriched in photosynthesis and carbon metabolism. Thus, the vast majority of enzymes involved in the Calvin cycle and photosynthesis were mapped to further investigate the potential function of Khib in rice (Fig. 5).

A surprising number of enzymes were found to be modified by 2-hydroxyisobutyrylation, including ribulose-1,5-bisphosphate carboxylase/oxygenase (Rubisco), glyceraldehyde 3-phosphate dehydrogenase (GAPDH), phosphoglycerate kinase (PGK), and fructose-1,6-bisphosphatase (FBP), as well as other key enzymes involved in carbon fixation, reduction, and regeneration. Throughout the entire photosystem in green plants, light-harvesting chlorophyll protein complex (LHC) contributes to the absorption and transfer of light energy. In rice leaves, 11 subunits of LHC (Lhca1–5 and Lhcb1–6) were found to be modified by 2-hydroxyisobutyrylation (Additional file 3: Table S2). Photosynthesis-associated proteins bearing Khib are widely distributed in cytochrome b6f complex, photosystem I and II, and ATP synthase (Fig. 5).

### Overlaps of Lysine 2-Hydroxyisobutyrylation, Acetylation, and Crotonylation in Rice

**Fig. 6:**
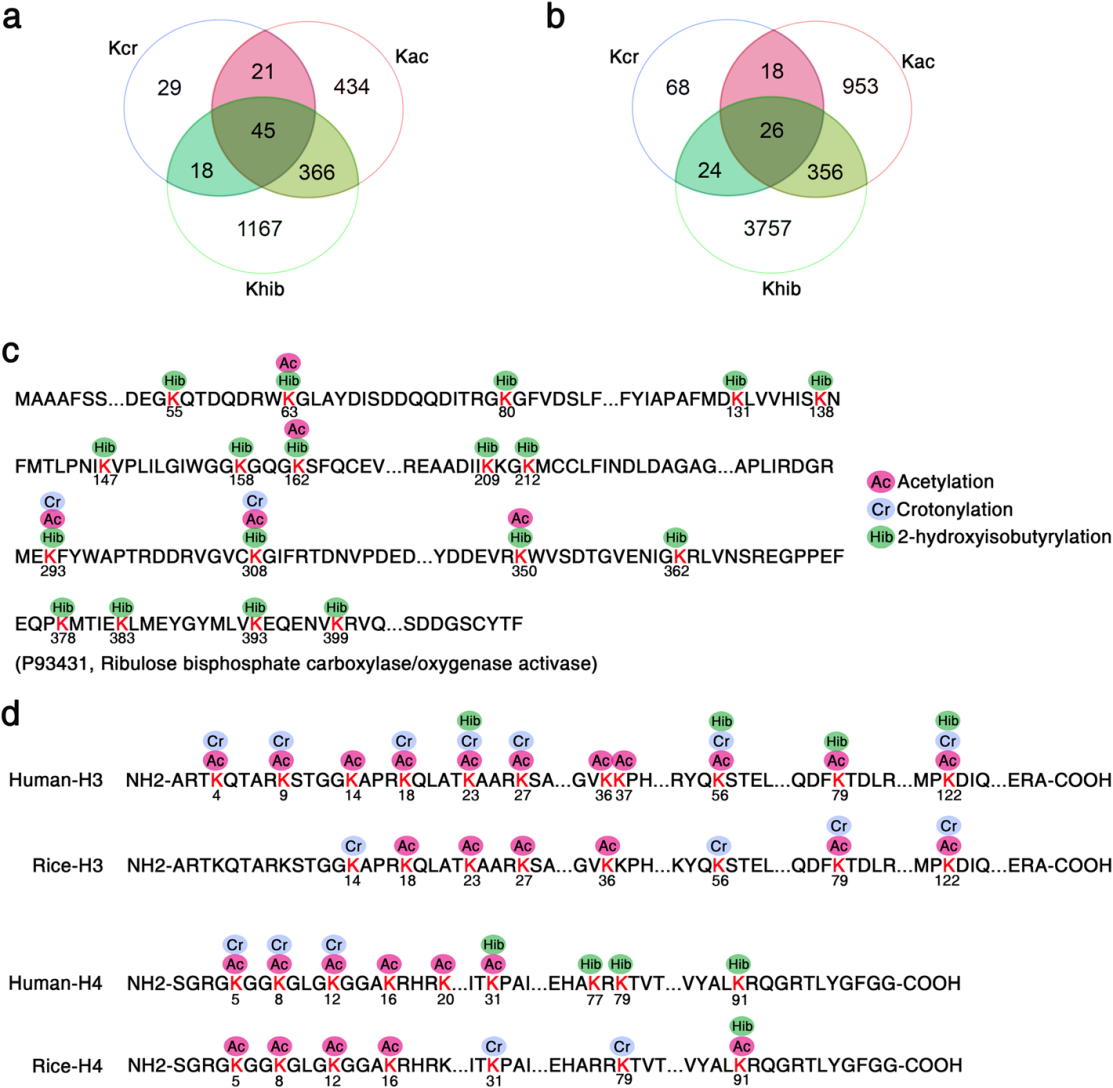
Comparison of the 2-hydroxyisobutyrylome with the lysine acetylome and lysine crotonylome in rice leaves. **a** The overlapped number of Khib proteins among Khib, Kac, and Kcr. **b** The overlapped number of Khib sites among Khib, Kac, and Kcr. **c** Khib, Kac, and Kcr sites on the representative protein Rubisco activase (P93431). **d** Comparison of histone H3 and H4 with Khib, Kac, and Kcr sites between rice and human

With the development of high-resolution MS techniques, an increasing number of novel sites and types of acylation have been identified in rice proteins, including Kac (Xiong et al. 2016; Xue et al. 2018), Khib (Meng et al. 2017), Ksucc (He et al. 2016), Kcr (Liu et al. 2018), Kbu (Lu et al. 2018), and Kma (Mujahid et al. 2018). Based on our previous proteome profiling of Kac and Kcr in rice seedling leaves (ProteomeXchange dataset identifier PXD004870 and PXD010376; *Oryza sativa* variety *Nipponbare*) (Xue et al. 2018; Liu et al. 2018), we performed a comparative analysis with the Khib proteins identified in this study (Fig. 6).

The number of Khib sites and proteins reported in this study is higher than those in the acetylome and crotonylome identified in seedling leaves (Fig. 6a). The number of overlapped Khib proteins is 411 and 63 for Kac and Kcr, respectively. Forty-five of the overlapped proteins for the three acylations are mainly associated with metabolic processes, oxidation reduction, and photosynthesis. In addition, 1167 (73.1%) of the 1569 modified proteins are only 2-hydroxyisobutyrylated. Among the acyl-modified sites, we identified 26 lysine sites that bear all three PTMs (Fig. 6b). The representative protein ribulose bisphosphate carboxylase/oxygenase activase (Rubisco activase, P93431), showed overlap of Khib, Kac, and Kcr (Fig. 6c; Additional file 1: Fig. S2a).

PTMs on histones are associated with diverse functions, including gene transcriptional activity, chromosome assembly, the cell cycle, and DNA damage and repair (Chen and Tian 2007; Liu et al. 2017; Sabari et al. 2017; Wang et al. 2017). Khib, as a new type of histone mark first reported in human and mouse cells, has been found to be catalyzed by histone acetyltransferases (Huang et al. 2018b; Dai et al. 2014). In this study, we identified 20 Khib sites in core histones (H2A/H2B/H4), among which only one site (K91) was identified in histone H4, but not in histone H3 (Fig. 6d; Table 1; Additional file 1: Fig. S2b). This is consistent with the Western blotting results (Fig. 1a). Nevertheless, seven histone Khib sites (H3K23/K56/K79/K122 and H4K31/K77/K79) in humans were not identified in this study. Compared to the histone Kac and Kcr sites derived from proteome-wide acylation analyses in our previous studies (Liu et al. 2018; Xue et al. 2018), fewer Khib sites were detected in the most conserved histones H3 and H4. Unlike acetylation, 2-hydroxyisobutyrylation and crotonylation, as two new types of histone acylations reported in recent years, require further research to expand the proteome profiles of rice.

**Table 1:** Lysine 2-hydroxyisobutyrulated sites identified in histones

Protein accession	Position	Modified sequence	Histone description	Mass error [ppm]
Q6ZL42	121	K(0.976)AGGSAK(0.024)AAAGD	H2A.2	−0.42403
Q6ZL43	14	AIGAGAAK(1)K	H2A.1	0.15735
Q94E96	137	TAEK(1)AAAAGK	H2A.5	−0.281
Q94E96	11	MDAAGAGAGGK(1)LK	H2A.5	0.17246
Q94E96	133	K(0.77)TAEK(0.23)AAAAGK	H2A.5	−0.98919
Q8S857	25	AAADK(0.004)DK(0.996)DRK	H2A variant 2	2.6074
Q8S857	13	GLLAAK(1)TTAAK	H2A variant 2	−1.4744
Q84NJ4	14	AIGSSAAK(1)K	H2A.3	−0.70687
Q9LGH8	67	SVETYK(1)IYIFK	H2B.8	1.4873
Q9LGH8	75	VLK(1)QVHPDIGISSK	H2B.8	0.13234
Q9LGH8	61	K(1)SVETYK	H2B.8	0.61662
Q9LGH8	108	LAGESAK(1)LAR	H2B.8	0.77039
Q9LGH8	26	AEK(1)APAGK	H2B.8	0.76614
Q9LGH8	137	LVLPGELAK(1)HAVSEGTK	H2B.8	−0.78709
Q9LGH8	145	HAVSEGTK(1)AVTK	H2B.8	−0.72084
Q9LGH8	72	IYIFK(1)VLK	H2B.8	−0.89643
Q9LGH8	23	KPAEEEPAAEK(1)AEK	H2B.8	−0.20801
Q943L2	18	KPVEEK(1)AEK	H2B.11	−0.032173
Q9LGH4	108	LAAEAAK(1)LAR	H2B.6	1.7336
Q6F362	23	KPAEEEPAAEK(1)APAAGK	H2B.9	0.78845
Q7XUC9	92	TVTAMDVVYALK(1)R	H4	−0.57386

The number in parenthesis indicates the localization probability of Khib modification

### Protein-Protein Interaction (PPI) Networks of Khib Proteins in Rice

**Fig. 7:**
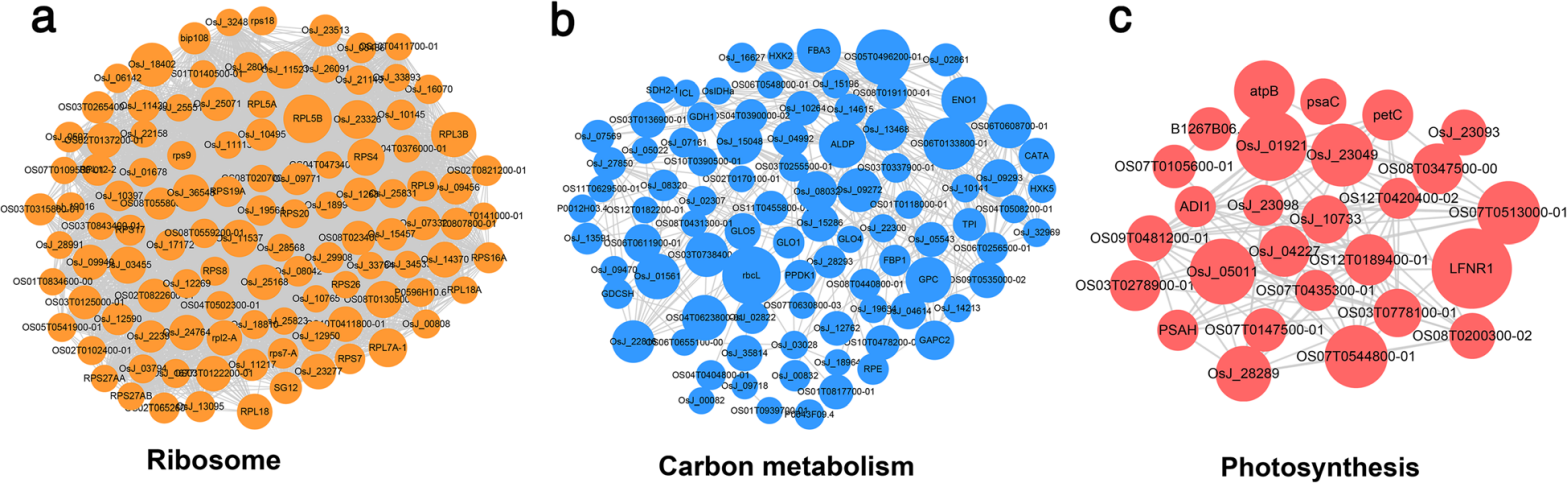
The three most enriched clusters in the PPI networks of Khib proteins. The network of Khib protein interactions (listed with protein ID names) as analyzed using Cytoscape. **a** Ribosome. **b** Carbon metabolism. **c** Photosynthesis. The size of the dots represents the number of Khib sites in each figure

To further evaluate the correlation of 2-hydroxyisobutyrylated proteins in rice, we constructed a protein-protein interaction network using the STRING database and Cytoscape software (Smoot et al. 2011). We fetched all interactions that had a combined score ≥ 0.9 (highest confidence) and identified 703 Khib proteins as nodes connected by 9834 interactions obtained from the STRING database (Version 11.0; Additional file 4: Table S3). A comprehensive PPI network of 2-hydroxyisobutyrylated proteins in rice is displayed in Fig. S6. Using a clustering algorithm performed with the MCODE tool, 37 clusters of Khib proteins were retrieved from the PPI network (Additional file 4: Table S3). Based on the KEGG pathway enrichment analyses, the most abundant interaction network was identified as ribosome sub-networks, containing 218 ribosome-associated proteins with Khib modification (Fig. 7a). The photosynthesis and carbon metabolism sub-networks consist of 180 and 54 metabolism-associated proteins, respectively (Fig. 7b, c).

**Fig. 8:**
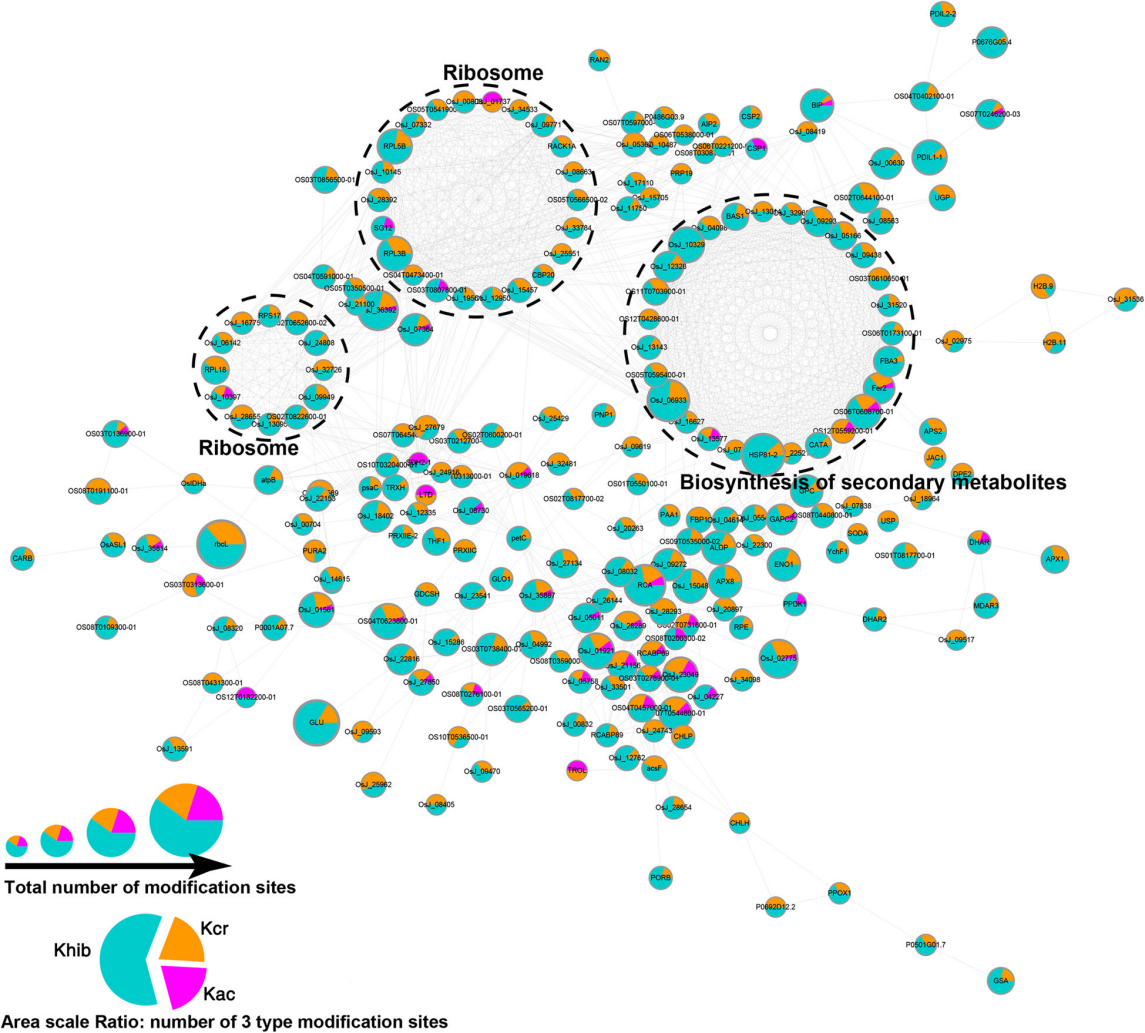
The crosstalk PPI network of the overlapped proteins with Khib modification

To investigate the effect of different acylations on protein function, we further analyzed the crosstalk PPI network between Kac, Kcr, and Khib modifications (Fig. 8). The crosstalk network of the 230 overlapped modified proteins (containing at least two modifications) showed that ribosome complex and biosynthesis of secondary metabolites sub-networks were highly connected.

## Discussion

The catalogue of lysine acylations has been extended over the past half century, largely due to the application of high-sensitivity mass spectrometry and the development of high-specificity antibodies (Huang et al. 2014). Khib is a newly discovered PTM, first reported in human and mouse cells, and it plays a critical role in the regulation of chromatin functions (Dai et al. 2014). In the present study, we performed a global proteome identification of Khib in rice seedling leaves. A total of 4163 Khib sites on 1596 proteins were identified for expansion of the lysine 2-hydroxyisobutyrylome in plants, profiling the regulatory roles of Khib in the growth and development of rice.

Compared to the previously reported 2-hydroxyisobutyrylome in developing rice seeds (Meng et al. 2017), relatively few overlapped proteins with Khib sites and the fewer number of identified sites were observed in our dataset (Table 2). It is probably due to the completely different tissues with different types and numbers of proteins. Between the 2-hydroxyisobutyrylomes in rice seedling leaves and developing seeds, only 18.35% proteins and 8.65% lysine sites with 2-hydroxyisobutyrylation overlap. In addition, similar results have been reported for the proteome profiles of other PTMs in the same species, even in the same organs (Meng et al. 2018; Xue et al. 2018). It has been speculated that distinct sampling stages or organs lead to differences in proteome identification by the same LC-MS/MS method. Furthermore, the Khib sites and modified proteins identified in developing rice seeds or seedling leaves contribute to the expansion of the entire rice 2-hydroxyisobutyrylome. Interestingly, although the 2-hydroxyisobutyrylomes of developing rice seeds and seedling leaves exhibit low levels of overlapping, similar conserved motifs are observed around the Khib sites. The motif patterns exhibit a bias for polar negatively charged amino acids, i.e., D and E, such as EK_hib_, DK_hib_, K_hib_D, K_hib_E, and D_XX_K_hib_ (Meng et al. 2017). Like 2-hydroxyisobutyrylation, a preference for amino acids D and E around the modified lysine residue is also observed in the crotonylomes of rice (Liu et al. 2018), tobacco (Sun et al. 2017), zebrafish (Kwon et al. 2018) and humans (Xu et al. 2017) suggesting that 2-hydroxyisobutyrylation and crotonylation are probably catalyzed by the same enzyme system. In contrast, only one common motif pattern (K_XXXXXXXX_K_PTM_) was identified in rice 2-hydroxyisobutyrylome and acetylome (Meng et al. 2018; Xue et al. 2018), which has not been reported as a Khib or Kac motif in other plants.

**Table 2:** Overlapped Khib sites and proteins between rice seedling leaves and developing seeds

Organs	Sampling dates	No. of Khib proteins	No. of Khib sites	Overlap (proteins/sites)	Reference
**leaves**	3-week seedlings	1596	4163	–	This study
**developing seeds**	15 days post anthesis	2512	9916	745/1219 (18.35%/8.65%)	(Meng et al. 2017)

In our dataset, 44% of the Khib proteins in rice are located in the chloroplast, presenting high correlation with photosynthesis and carbon fixation, which is consistent with the distribution of Kac and Kcr in rice leaves (Liu et al. 2018; Xiong et al. 2016; Xue et al. 2018). Photosynthesis is a pivotal process in green plants, converting solar energy into active chemical energy in the form of ATP and stable chemical energy in the form of sugars (Gest 2002). A large number of enzymes involved in photosynthesis and carbon metabolism were found to be 2-hydroxyisobutyrylated, including ATP synthase (subunit a/b/c and α/β/γ/ε), CO_2_ fixation enzyme (Rubisco), reductases (GAPDH, PGK), and other photosystem complexes (photosystem I and II, cyt b6/f, and FNR).

In *Arabidopsis thaliana*, acetylation at the K201 and K334 sites of the key enzyme in fixing atmospheric CO_2_, i.e., Rubisco, negatively regulates its activity and decreases CO_2_ fixation efficiency (Gao et al. 2016). We also identified Khib sites on the Rubisco in rice leaves, and found Kac, Kcr, and Khib co-modified at K293 and K308 of Rubisco activase, which facilitates Rubisco remodeling (Stotz et al. 2011). Moreover, PPI network analysis of Khib proteins in this study revealed numerous sub-networks involved in the ribosome complex, biosynthesis of secondary metabolites, and metabolic pathways, including a staggering proportion of proteins located in the chloroplast (Additional file 4: Table S3). These findings suggest that Khib plays important roles in modulating carbon fixation, energy transduction, and photosynthetic efficiency in plants.

Khib was first reported as a novel and active histone mark in human and mouse cells (Dai et al. 2014). As a new type of acylation, histone Khib neutralizes the positive charge of lysine, much like Kac or Kcr does, but the distinct structure of the modifier leads to a much larger change in size and distinct genomic distribution from those of histone Kac or Kcr (Dai et al. 2014). In our study, 21 Khib sites were identified on the core histones in rice, including H2A (8 sites), H2B (12 sites), and H4 (only one site, K91). Unexpectedly, Khib was not detected on histone H3.

In the study by Dai et al., 22 histone Khib sites were detected in HeLa cells, including H3K23/K56/K79/K122 and H4K31/K77/K79/K91, while 60 histone Khib sites were identified in mouse total testis cells (Dai et al. 2014). They proved that H4K8hib, like H4K8ac, is associated with transcriptional activity (Dai et al. 2014; Montellier et al. 2012). H4K91, the only overlapped Khib site on conserved histone H4 between our dataset and 2-hydroxyisobutyrylome in humans, is in the core domain of histones rather than on the NH_2_-terminal tail. Previous studies have revealed that variations in H4K91 result in abnormal DNA repair and silent chromatin formation (Ma et al. 1998; Ye et al. 2005). The acetylation of the only overlapped Khib site on conserved histone H4 (H4K91) has been found to restrain the reassembly of histone octamers by weakening H3/H4-H2A/H2B binding (Ye et al. 2005). However, more information regarding the regulatory function and mechanism of histone Khib in plants and animals is required.

## Conclusions

Lysine 2-hydroxyisobutyrylation is a novel protein post-translational modification initially reported in mammals and involved in the regulation of chromatin functions. This study comprehensively profiles the lysine 2-hydroxyisobutyrylome in rice leaves. Based on systematic proteomic analysis, we found that Khib occurs in proteins involved in biological functions and metabolic processes. Our results provide a better understanding of the diverse functions of lysine 2-hydroxyisobutyrylation in plants.

## Materials and Methods

### Rice Seedlings and Growth Conditions

Seedlings of *Oryza sativa* variety *Nipponbare* were grown in a growth chamber at 28 or 25 °C in 12 h light/12 h dark conditions. After 3 weeks of growth, seedling leaves without stems and sheaths were excised and immediately prepared for protein extraction.

### Western Blot Assay

The sample (100 mg leaf material) was ground by liquid nitrogen into powder and then transferred to a 2.0 mL centrifuge tube. Total proteins of the sample were prepared with extracting solution (50 mM Tris-HCl, 150 mM NaCl, 0.1% NP-40, 24% Urea, 1 mM DTT, and 1 mM protease inhibitor). The chloroplast proteins were extracted using Chloroplast Protein Extraction Kit (BestBio. BB-3179-1) following by the manufacturer’s instructions. Proteins were separated by 12% SDS-PAGE and transferred onto a PVDF membrane (Millipore). Antibody for pan-2-hydroxyisobutyryl lysine (PTM-801, PTM Biolabs) was used as the primary antibody (1:3000 dilution). Signals were detected using SuperSignal West Pico Plus chemiluminescent substrate (Thermo Scientific).

### Protein Extraction, Trypsin Digestion, and Affinity Enrichment

The sample (1 g leaf material) was ground by liquid nitrogen into powder and then transferred to a 5-mL centrifuge tube. Then, four volumes of lysis buffer (8 M urea, 1% Triton-100, 10 mM dithiothreitol, 1% protease inhibitor cocktail, 3 μM TSA and 2 mM EDTA) was added to the tissue powder, followed by sonication three times on ice using a high intensity ultrasonic processor (Scientz). The remaining debris was removed by centrifugation at 20,000 g and 4 °C for 10 min. Finally, the protein was precipitated with cold 20% TCA for 2 h at − 20 °C. After centrifugation at 12,000 g and 4 °C for 3 min, the supernatant was discarded. The remaining precipitate was washed with cold acetone three times. The protein was redissolved in 8 M urea and the protein concentration was determined with a BCA kit (P0011, Beyotime Bio) according to the manufacturer’s instructions.

For digestion, the protein solution was reduced with 5 mM dithiothreitol for 30 min at 56 °C and alkylated with 11 mM iodoacetamide for 15 min at room temperature in darkness. The protein sample was then diluted by adding 100 mM NH_4_HCO_3_ to provide a urea concentration of less than 2 M. Finally, trypsin was added at a 1:50 trypsin-to-protein mass ratio for the first digestion overnight and then at a 1:100 trypsin-to-protein mass ratio for a second 4 h-digestion.

To enrich the Khib modified peptides, tryptic peptides dissolved in IP buffer (100 mM NaCl, 1 mM EDTA, 50 mM Tris-HCl, 0.5% NP-40, pH 8.0) were incubated with pre-washed 2-hydroxyisobutyryl lysine antibody beads (PTM-804, PTM Biolabs) at 4 °C overnight with gentle shaking. The beads were then washed four times with IP buffer and twice with H_2_O. The bound peptides were eluted from the beads with 0.1% trifluoroacetic acid. Finally, the eluted fractions were combined and vacuum-dried. For LC-MS/MS analysis, the resulting peptides were desalted with C18 ZipTips (Millipore) according to the manufacturer’s instructions.

### LC-MS/MS Analysis

The tryptic peptides were dissolved in 0.1% formic acid (Solvent A), directly loaded onto a home-made reversed-phase analytical column (15 cm length, 75 μm i.d.). The gradient comprised an increase from 9% to 25% Solvent B (0.1% formic acid in 90% acetonitrile) over 38 min, an increase from 25% to 36% over 14 min, an increase to 80% over 4 min, then holding at 80% for the last 4 min, all at a constant flow rate of 300 nL/min on an EASY-nLC 1000 UPLC system.

The peptides were subjected to a nanospray ionization source followed by tandem mass spectrometry (MS/MS) on a Q Exactive™ Plus (Thermo Scientific) coupled online to the UPLC apparatus. The electrospray voltage applied was 2.0 kV. The *m/z* scan range was 350 to 1800 for full scan, and intact peptides were detected in the Orbitrap at a resolution of 70,000. Peptides were then selected for MS/MS using normalized collision energy (NCE) at 28% and the fragments were detected in the Orbitrap at a resolution of 17,500. A data-dependent procedure that alternated between one MS scan followed by 20 MS/MS scans with 15.0 s dynamic exclusion was used. Automatic gain control (AGC) was set at 5E4.

### Database Search

The resulting MS/MS data were processed using the Maxquant search engine (v1.5.2.8) (Cox and Mann 2008). Tandem mass spectra were searched against the UniProt *Oryza_sativa*_*Japonica* database (48,932 sequences) concatenated with a reverse decoy database. Trypsin/P was specified as the cleavage enzyme allowing up to four missing cleavages. The mass tolerance for precursor ions was set at 20 ppm in the first search and at 5 ppm in the main search, and the mass tolerance for fragment ions was set as 0.02 Da. Carbamidomethyl on Cys was specified as a fixed modification, and oxidation on Met, acetylation on the N terminal, and 2-hydroxyisobutyrylation on lysine were specified as variable modifications. False discovery rate (FDR) was adjusted to < 1% (Elias and Gygi 2007) and the minimum score for modified peptides was set at 40.

### Motif Enrichment, GO, and Pathway Analyses

Motif-x software (Chou and Schwartz 2011) was used for analysis of model sequences constituted by amino acids in specific positions of modifier-21-mers (10 amino acids upstream or downstream of acetylation sites) in all protein sequences. All the database protein sequences were used as background database parameters, with other parameters at default.

Proteins were classified by GO annotation (http://www.ebi.ac.uk/GOA/) into three categories: biological process, cellular compartment, and molecular function. For each category, a two-tailed Fisher’s exact test was applied to test the enrichment of the differentially expressed protein against all identified proteins. Correction for multiple hypothesis testing was carried out using standard FDR control methods. GO with a corrected *p*-value < 0.05 is considered significant.

The Encyclopedia of Genes and Genomes (KEGG) database was used to identify enriched pathways by a two-tailed Fisher’s exact test to test the enrichment of the identified modified protein against all proteins in the UniProt Oryza_sativa_Japonica database. Correction for multiple hypothesis testing was carried out using standard FDR control method, and the pathway with a corrected p-value < 0.05 was considered to be significantly enriched. These pathways were classified into hierarchical categories according to the KEGG website (http://www.genome.jp/kegg/pathway.html).

### Khib PPI Network Analyses

The STRING database was used to annotate the functional interactions of all the identified acetylated proteins by calculating their confidence score. Only high confidence interactions (> 0.9) in the STRING database (Version 11.0) were fetched for the analysis. Cytoscape software (Version 3.6.1) was used to visualize the interaction network. To identify sub-clustering groups from the protein interaction network generated by STRING, Molecular Complex Detection (MCODE) was utilized through vertex weighing by local neighborhood density and outward traversal from a local dense node (Bader and Hogue 2003).

## Supplementary information

**Additional file 1 Fig. S1.** Quality control validation of MS data and distribution of Khib peptides. **Fig. S2.** Representative MS/MS spectra of Khib-modified peptides in rice. **Fig. S3.** Western blotting analysis of chloroplast proteins in rice seedling leaves. **Fig. S4.** GO-based enrichment analysis of identified Khib proteins. **Fig. S5.** KEGG pathway enrichment analysis of rice Khib proteins. **Fig. S6.** PPI network of all identified Khib proteins.

**Additional file 2 Table S1.** Annotations of all identified 2-hydroxyisobutyrylated proteins and the modified sites in rice seedlings.

**Additional file 3 Table S2.** Identified Khib subunits in the chlorophyll protein complex.

**Additional file 4 Table S3.** Node and network information for the PPI network.

## Data Availability

The datasets used and analyzed during the current study are available from the corresponding author on reasonable request. The mass spectrometry proteomics data have been deposited to the ProteomeXchange Consortium via the PRIDE partner repository with the dataset identifier PXD018778.

## Abbreviations

Khib: Lysine 2-hydroxyisobutyrylation
LC-MS/MS: Liquid chromatography-tandem mass spectrometry
PTM: Post-translational modification
KAT: Lysine acetyltransferases
KDAC: Lysine deacetylases
SDS-PAGE: Sodium dodecyl sulfate polyacrylamide gel electrophoresis
PPI: Protein-protein interaction
KEGG: Kyoto Encyclopedia of Genes and Genomes
GO: Gene Ontology
MCODE: Molecular Complex Detection
NCE: Normalized collision energy
AGC: Automatic gain control
FDR: False Discovery Rate
