# Supplementary material for: Proteome-Wide Analyses Reveal the Diverse Functions of Lysine 2-Hydroxyisobutyrylation in Oryza sativa
Source: Rice (N Y). 2020 Jun 5;13:34. doi: 10.1186/s12284-020-00389-1 (PMC7310055; doi:10.1186/s12284-020-00389-1)
Supplement: Supplementary file 1 — Additional file 1 Fig. S1. Quality control validation of MS data and distribution of Khib peptides. Fig. S2. Representative MS/MS spectra of Khib-modified peptides in rice. Fig. S3. Western blotting analysis of chloroplast proteins in rice seedling leaves. Fig. S4. GO-based enrichment analysis of identified Khib proteins. Fig. S5. KEGG pathway enrichment analysis of rice Khib proteins. Fig. S6. PPI network of all identified Khib proteins. [file 12284_2020_389_MOESM1_ESM.pdf]

Proteome-wide Analyses Reveal the Diverse Functions of Lysine 2-Hydroxyisobutyrylation in *Oryza sativa*

Chao Xue[1,2#], Zhongying Qiao[3#], Xu Chen[1,2], Penghui Cao[3], Kai Liu[1,2], Shuai Liu[1,2], Lu Ye[1,2], Zhiyun Gong[1,2*]

1: Jiangsu Key Laboratory of Crop Genetics and Physiology/ Key Laboratory of Plant Functional Genomics of the Ministry of Education/ Jiangsu Key Laboratory of Crop Genomics and Molecular Breeding, Agricultural College of Yangzhou University, Yangzhou 225009, China

2: Jiangsu Co-Innovation Center for Modern Production Technology of Grain Crops, Yangzhou University, Yangzhou 225009, China

3: Suzhou Academy of Agricultural Sciences, North of Wangting Town, Suzhou 215128, China

#: These authors contributed equally to this work.

*For correspondence (e-mail: zygong@yzu.edu.cn)

The PDF file includes:

Figure S1. Quality control validation of MS data and distribution of Khib peptides.

Figure S2. Representative MS/MS spectra of Khib-modified peptides in rice.

Figure S3. Western blotting analysis of chloroplast proteins in rice seedling leaves.

Figure S4. GO-based enrichment analysis of identified Khib proteins.

Figure S5. KEGG pathway enrichment analysis of rice Khib proteins.

Figure S6. PPI network of all identified Khib proteins.

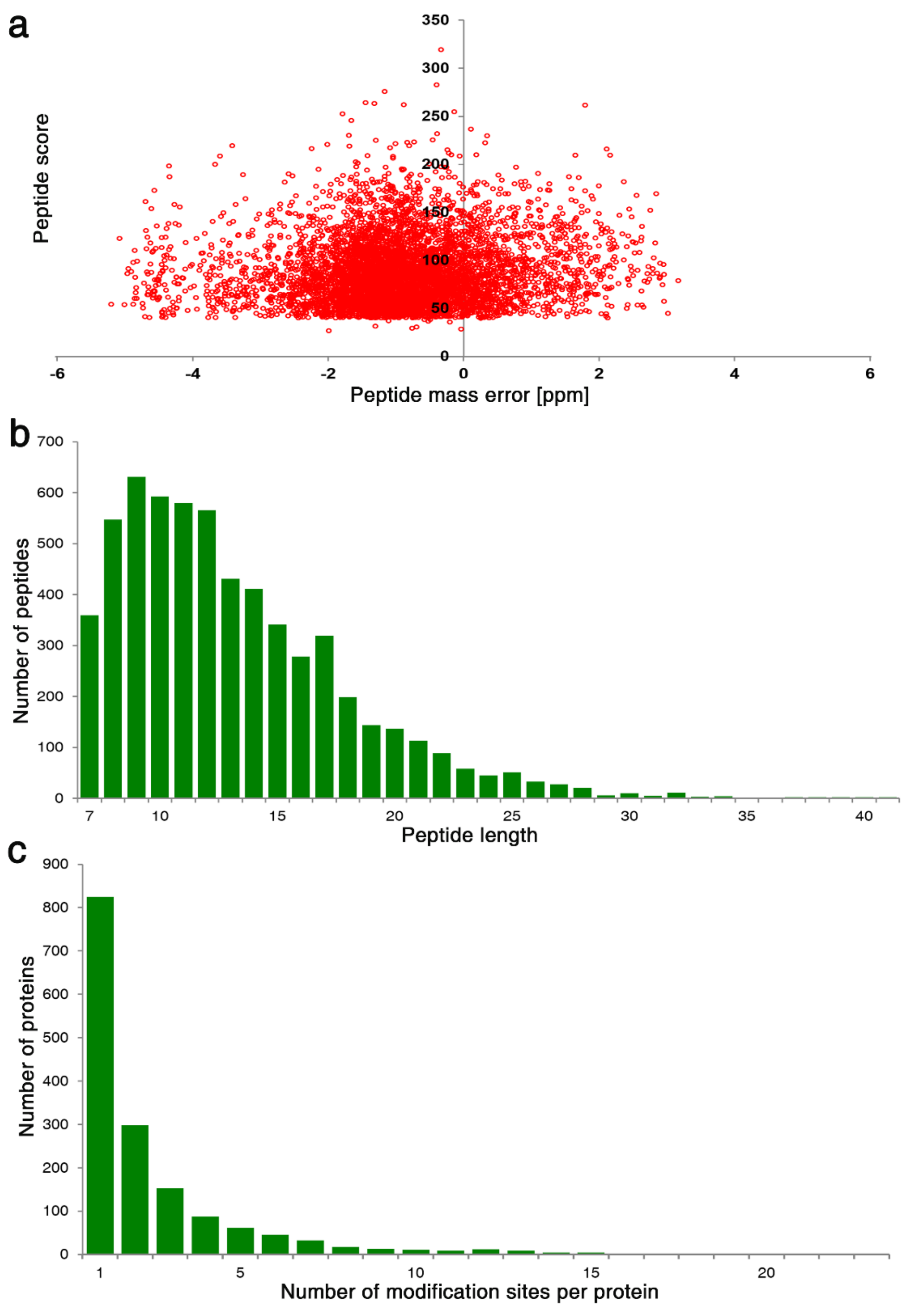

Figure S1. Quality control validation of MS data and distribution of Khib peptides. (a)The mass errors of peptides with Khib sites. (b) The lengths of all identified peptides. (c) The number of Khib sites per protein.

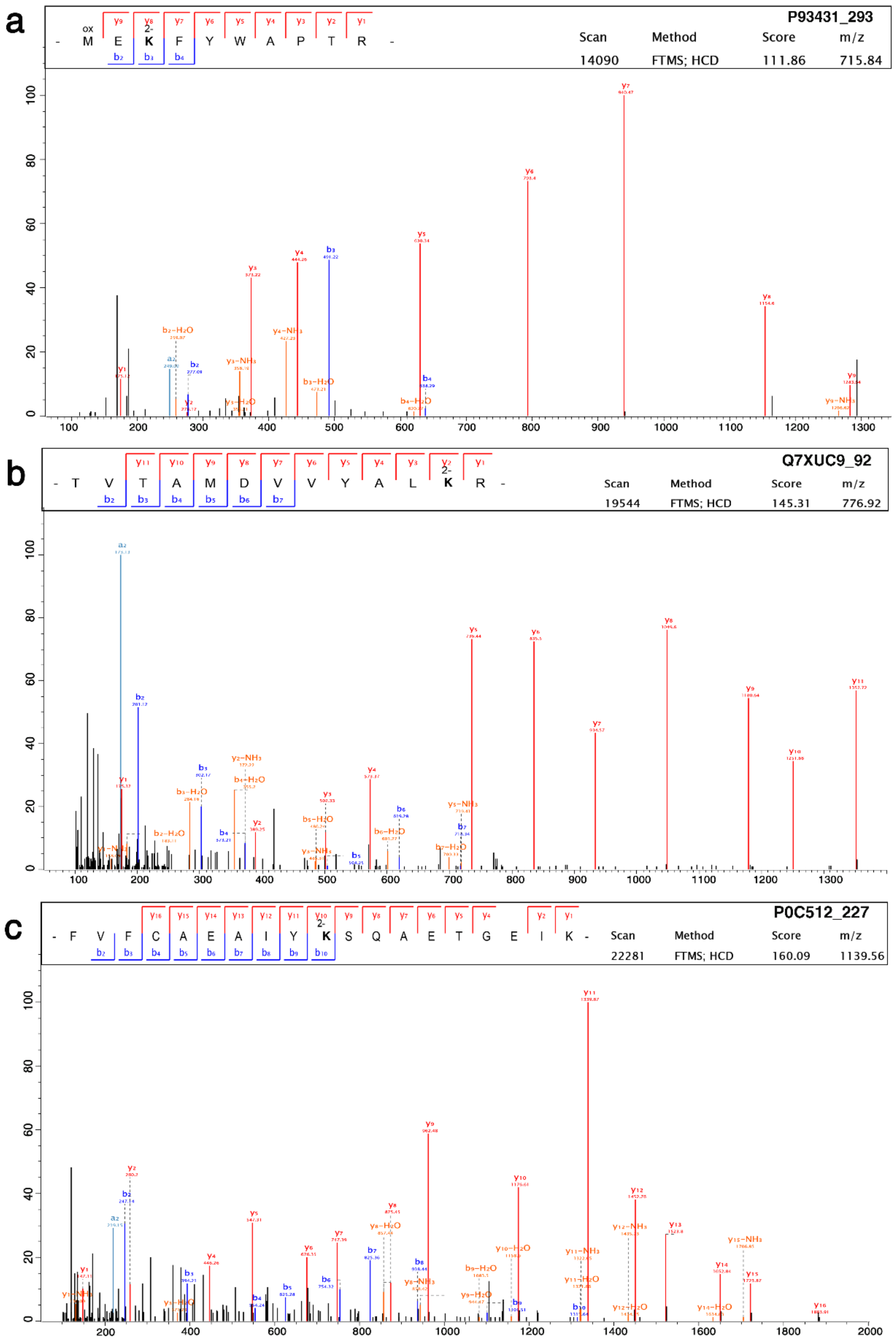

Figure S2. Representative MS/MS spectra of Khib-modified peptides in rice. **(a)** MS/MS spectrum of MEK$_{hib}$FYWAPTR in Rubisco activase (P93431). **(b)** MS/MS spectrum of TVTAMDVVYALK$_{hib}$R in histone H4 (Q7XUC9). **(c)** MS/MS spectrum of FVFCAEAIYK$_{hib}$SQAETGEIK in Rubisco (P0C512).

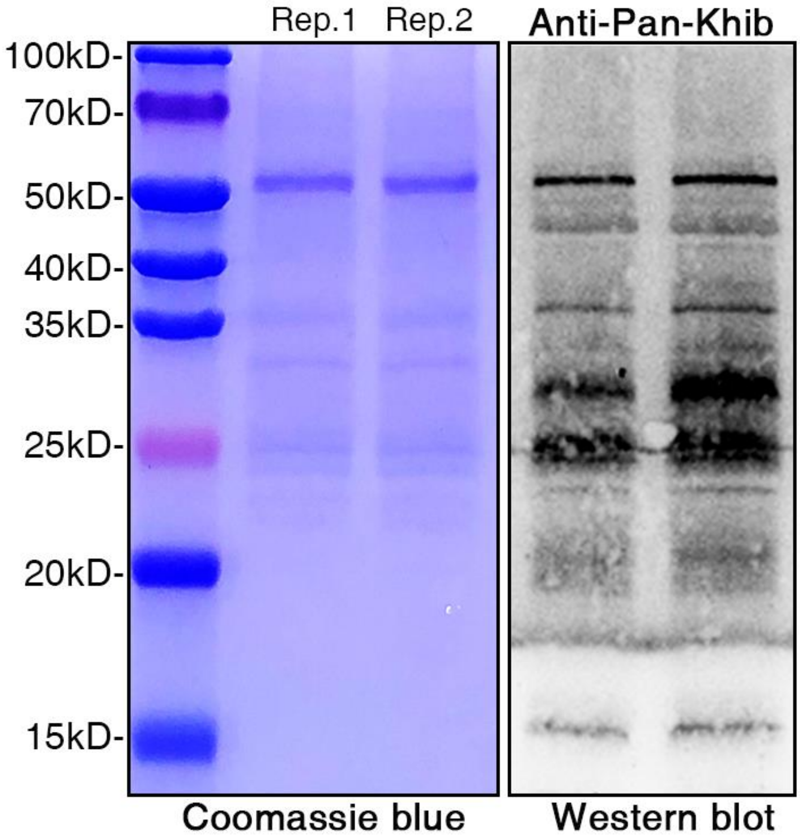

Figure S3. Western blotting analysis of chloroplast proteins in rice seedling leaves.

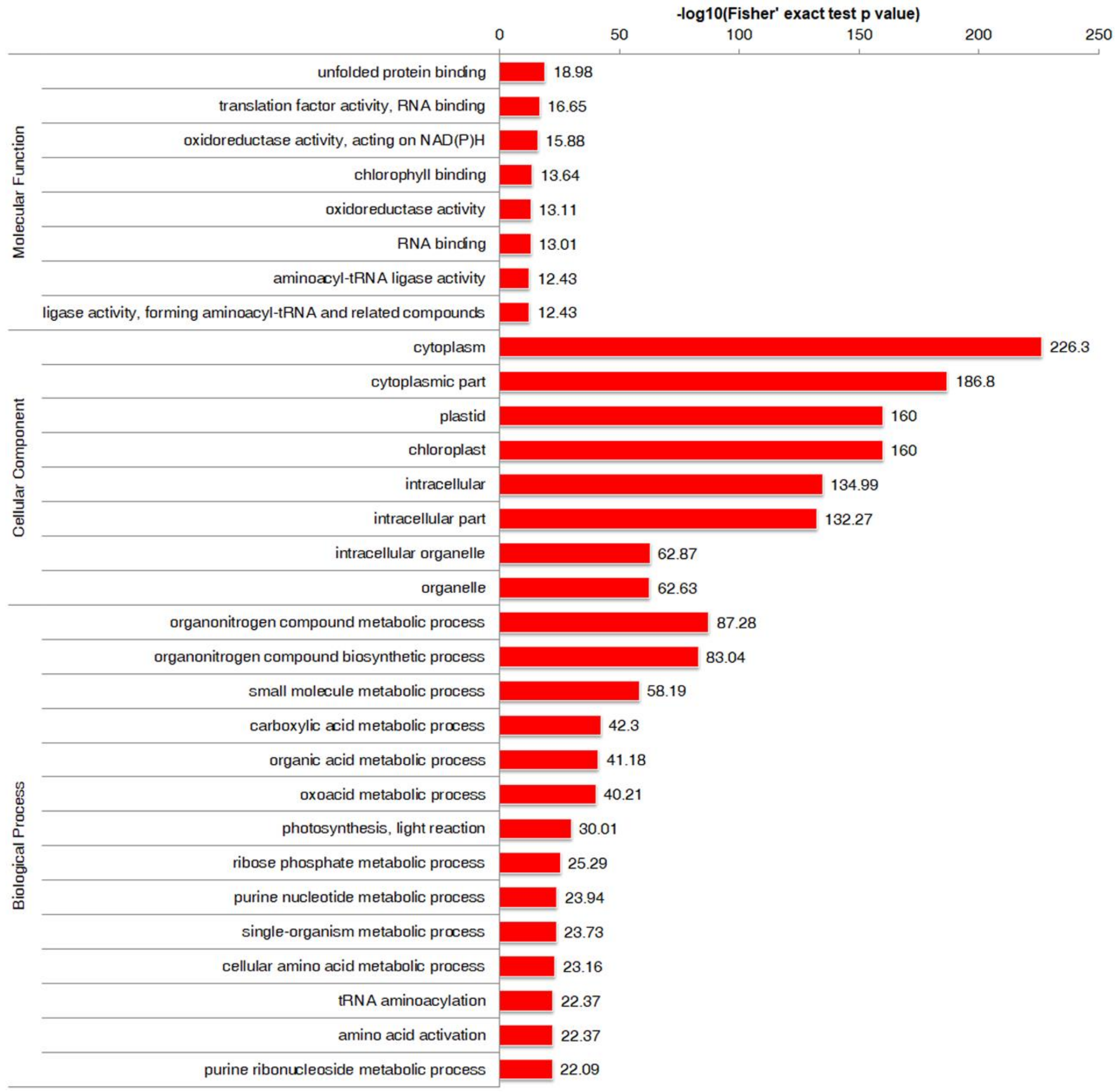

Figure S4. GO-based enrichment analysis of identified Khib proteins. The value of -log10 (Fisher's test p value) is shown.

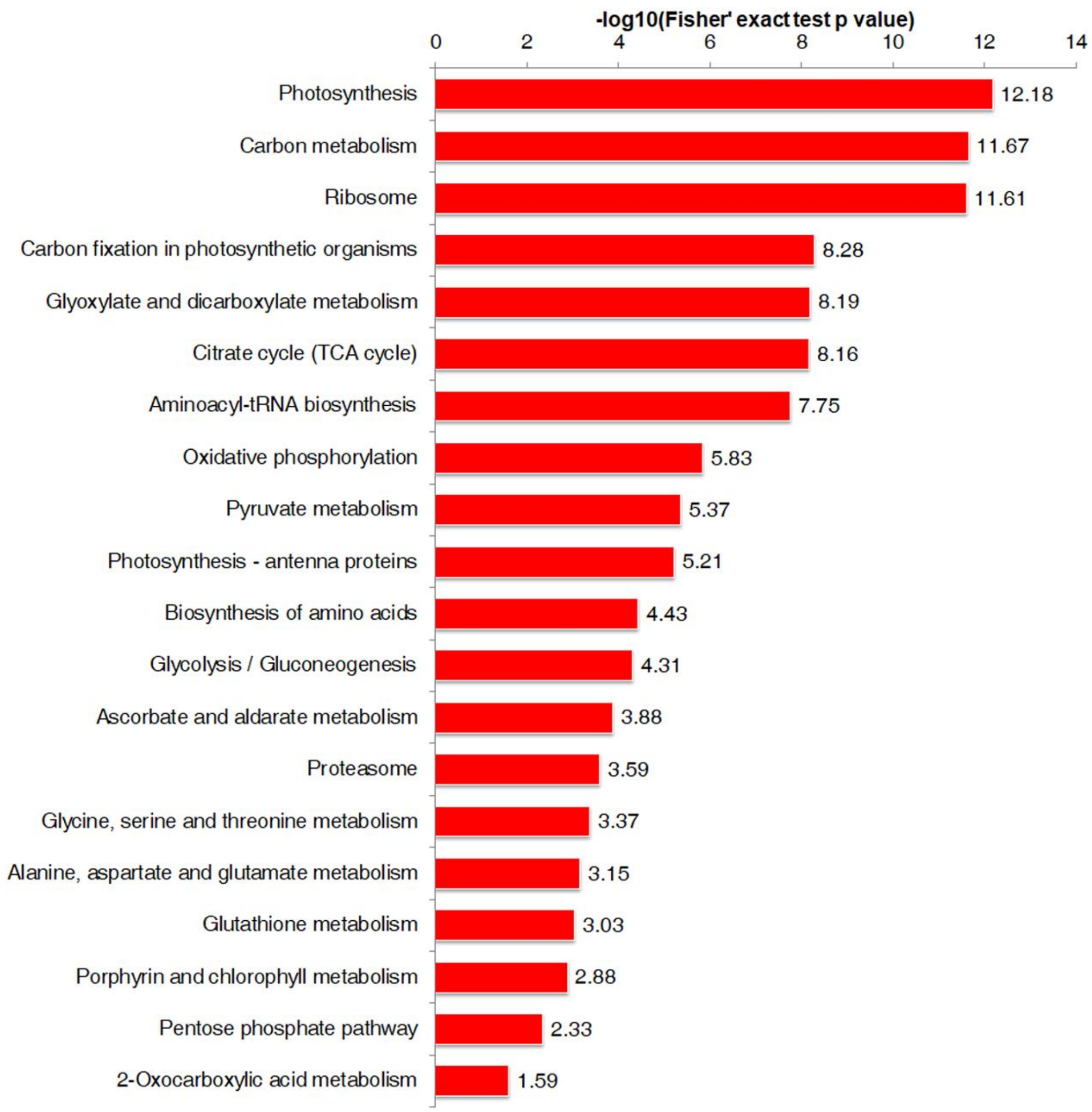

Figure S5. KEGG pathway enrichment analysis of rice Khib proteins. The value of -log10 (Fisher's test p value) is shown.

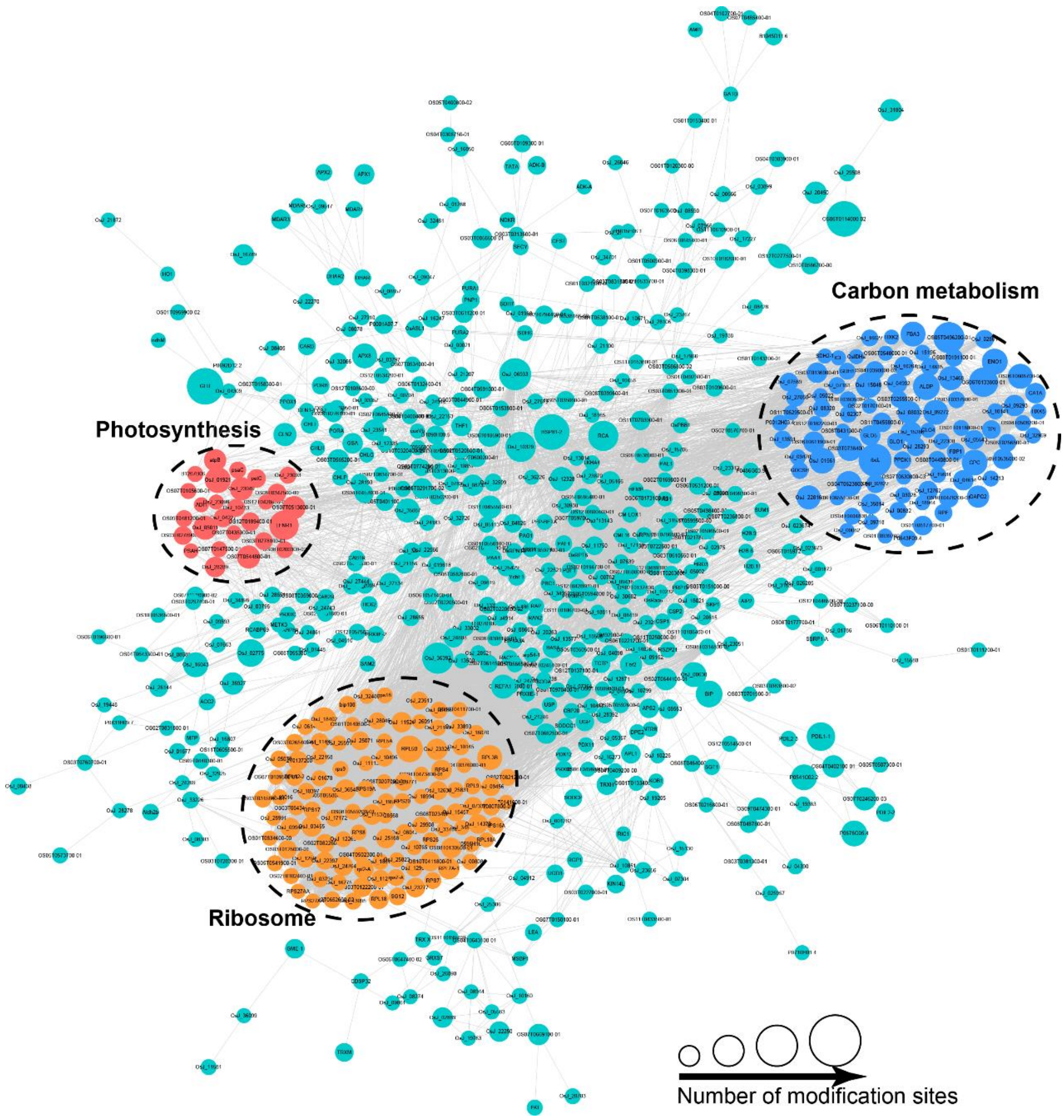

Figure S6. PPI network of all identified Khib proteins. The network of Khib protein interactions (listed with protein ID names) as analyzed using Cytoscape. The size of the dots represents the number of Khib sites.
